# Predictors for the duration of breastfeeding among ethiopia women of childbearing age with babies; application of accelerate failure time and parametric shared frailty models

**DOI:** 10.1186/s40795-022-00601-z

**Published:** 2022-09-22

**Authors:** Getahun Mulugeta, Dagne Tesfaye, Awoke Seyoum Tegegne

**Affiliations:** 1 Department of Statistics, Debre Berhan University, Debre Berhan, Ethiopia; 2 Department of Statistics, Bahir Dar University, Bahir Dar, Ethiopia

**Keywords:** Survival data analysis, Accelerated failure model, Censoring, Duration of breastfeeding

## Abstract

**Introduction:**

Duration of breastfeeding is the length of the time that infants who were initially breastfed continue to receive breast milk until weaning. The duration of breastfeeding is important for a child's health, growth, and development. However, the duration of breastfeeding decreases from time to time and further leads children to be exposed to malnutrition (stunting, wasting, and weight loss). Children who did not get enough breastfeeding are also exposed to different diseases. Previous studies used a simple survival model and didn’t see the shared frailty model on the variable of interest. Therefore, the current study aimed to investigate the factors affecting the duration of breastfeeding among Ethiopian women of reproductive age with babies.

**Methods:**

A cross-sectional study design was conducted on 15,400 women of childbearing age with babies in nine regional states and two city administrations. The data source for the analysis was the 2016 EDHS data. The Cox-proportional hazard model, AFT, and parametric shared frailty models were conducted for the current investigation. Weibull-gamma shared frailty model was in favor of others for current data analysis.

**Results:**

Among the covariates, women living in urban area (Φ = 0.96; 95% CI; (0.94,0.97); *p*-value = 0.001), non-educated women(Φ = 1.03; 95% CI; (1.00,1.06); *p*-value = 0.039), primary educated women (Φ = 1.13; 95% CI; (1.11,1.15); *p*-value < 0.001), age of a child (Φ = 0.99; 95% CI; (0.76.0.99); *p*-value < 0.001) and non-smoker mothers (Φ = 1.60; 95% CI; (1.57, 1.63); *p-*value < 0.001),birth interval between 2–3 years(Φ = 1.02; 95% CI;(1.09, 1.25, *p*-value = 0.027), birth interval, > 3 years(Φ = 1.28; 95% CI; (1.06, 1.43); *p*-value < 0.01 significantly affected the duration of breastfeeding. The median survival time of breastfeeding of women of reproductive age with babies considered under study was 23.4 months. Clustering had a significant effect on the variable of interest.

**Conclusion:**

Residence area, level of education, age of the child, smoking status of women, and birth interval of successive birth significantly affected the duration of breastfeeding in the current investigation. Hence, the health staff should conduct health-related education for young women, educated women, urban women, smoker women, and women with a shorter interval of birth to increase the women's attitude and awareness towards the use of long-duration of breastfeeding.

## Introduction

Breastfeeding is the act of breast milk transference from the mother to the infant. Breastfeeding is a unique source of nutrition that plays an important role in the growth, development, and survival of infants [1]. It satisfies a baby’s emotional needs and increases bonding between mother and infant, and it is considered to be physiologically, psychologically, and immunologically important [2]. Breastfeeding reduces the incidence of meningitis, malaria, asthma, respiratory diseases (such as pneumonia), ear infection, diarrhea, and urinary tract infection [3, 4].Breastfeeding also helps to reduce age-related disease (severe acute malnutrition (kwashiorkor) [5].Breastfeeding of an infant is not only crucial for optimal growth and development but also important for determinants of future physical and mental well-being because of the rapid growth and development of organs and tissues during the first year of life [6].

However, the duration of breastfeeding is declining in almost all parts of the world despite its nutritional and immunological benefits [7–10].An estimated number of 820,000 under-five children died because of the short duration of breastfeeding [11, 12].

In Ethiopia, the duration of breastfeeding decreases from 74 to 64% for infants of age group (0–1 month) and from 74 to 36% for infants of age group (4–5 months) [13]. The duration of breastfeeding decreases from 91 to 76% among children of the age group (18–23 months) [14]. Hence, the duration of breastfeeding is decreasing significantly and becomes below the WHO’s and UNICEF’s recommendations in the study area [15].

An insignificant number of researches are conducted on the issue concerned here, and most of the research conducted previously considered a simple survival model without considering the shared frailty term [16]. This gap became a motivation for the current investigation. To overcome, this gap, in the current study, the duration of breastfeeding of women were clustered by the region by introducing the shared frailty term in the usual survival model. The shared frailty term was also added to account for investigating the correlation that existed among the cluster of unobservable random effects. Hence, the current study used a parametric shared frailty model and accelerated failure time (AFT) models like Weibull, exponential, log-logistic, and log-normal distributions to compare and get the best model which fits the duration of breastfeeding of women data appropriately.

For the effective policy formulation and motivation of women for a longer duration of breastfeeding, it is crucial to study the various socio-economic and demographic factors affecting the duration of breastfeeding. Therefore, the objective of the current investigation was to identify socio-demographic, economic, and health-related factors affecting the duration of breastfeeding among Ethiopian women of reproductive age with babies.

## Methods and materials

### Study area and population

The current study was conducted across the country, Ethiopia, which consists of nine regions and two city administrations namely; Tigray, Afar, Amhara, Oromia Somali, Benishangul-Gumuz, the Old Southern Nations Nationalities and Peoples (SNNP), Gambela and Harari regional states and two city administrations, namely Addis Ababa and Dire Dawa. The study population under the current investigation consists of all women of childbearing age.

#### Source of data

The source of data for this study was reports of the Ethiopian Demographic and Health Survey (EDHS, 2016) which is obtained from the Central Statistical Agency (CSA) collected from January 18, 2016, to 27 June 2016. It is the fourth survey conducted in Ethiopia as part of the worldwide DHS project. The 2016 EDHS is designed to provide estimates for the health and demographic variables of interest.

### Sampling procedures and sample size understudy

Administratively, regions in Ethiopia are divided into zones, and zones, into administrative units, woredas. Each woreda is further subdivided into the lowest administrative unit, Kebele. Furthermore, each kebele was subdivided into census enumeration areas (EAs) or clusters. The 2016 EDHS sample was selected using a stratified, two-stage cluster sampling design (EDHS, 2016). Each region was stratified into urban and rural areas, yielding 21 sampling strata. Clusters are the sampling units for the first stage. The sample included 645 clusters (202 in urban areas and 443 in rural areas). Then, a fixed number of 28 households per cluster were selected with an equal chance of selection criteria from the newly created household listing. A total of 18,008 households were selected for the sample, of which 17,067 were interviewed. Among the interviewed households, 15,683 eligible women of reproductive age with babies were identified for individual interviews. Hence, this study considered the total number of 15,400 women of reproductive age with babies.

#### Inclusion criterion

All women of reproductive age (15–49 years) with babies were eligible for the current investigation.

### Variables in the study

#### The response variable

The duration of breastfeeding was considered as the dependent variable and defined as the period between the date of the beginning of breastfeeding and the time to stop breastfeeding before the end of the study.

$$\mathrm{Status }=\{\begin{array}{c}0 \ if\ censored\ (women\ still\ breastfeed\ until\ the\ end\ of\ the\ study)\\ 1\ if\ the\ event\ (women\ stopped\ breastfeed\ before\ the\ end\ of\ the\ study)\end{array}$$

#### Explanatory variables

The predictor variables considered under current investigation are indicated in Table1, in the results section.

**Table 1:** Baseline characteristics of participants with their duration of breastfeeding status

Variable	Categories	Frequency	Status	
		Total	Censored	event
Place of Residence	Rural	10,124(65.7%)	3452(34.1%)	6672(65.9%)
	urban	5276(34.3%)	800(15.2%)	4476(84.8%)
Wealth Index	Poor	6564(42.6%)	2269(34.6%)	4295(65.4%)
	Meddle	2798(18.2%)	749(26.8%)	2049(73.2%)
	Rich	6038(39.2%)	1234(20.4%)	4804(79.6%)
Employment status	Unemployed	10,072(65.4%)	2789(27.7%)	7283(72.3%)
	Employed	5328(34.6%)	1463(27.5%)	3865(72.5%)
Women education	no education	4986(32.4%)	793(28.7%)	1970(71.3%)
	Primary	7651(49.7%)	1875(37.6%)	3111(62.4%)
	Second. &above	2763(17.9%)	1584(20.7%)	6067(79.3%)
Sex	Male	5609(36.4%)	2280(40.6%)	3329(59.4%)
	Female	9791(63.6%)	1972(20.1%)	7819(79.9%)
Smoking status	no	14,188(92.1)	3958(27.9%)	10,230(72.1%)
	yes	1212(7.9)	294(24.3%)	918(75.7%)

### Methods of analysis and models used in the current investigation

In this study, SAS software (Version 9.4) was used for statistical data analysis. Semi-parametric methods such as Cox proportional hazards model were used for multivariable analysis. A comparison of the survival curve for each covariate was also conducted using the Kaplan–Meier method. The accelerated failure time model was also used to measure the direct effect of the explanatory variables on the survival time instead of a hazard. A shared frailty model was used to model the degree of correlation within groups assuming that all individuals in a given cluster share the same frailty; i.e., the subjects within a group are correlated because they share common frailty [17–19].

### Controlling and testing for the existence of confounding effects and effect modifiers

The existence of confounding was checked and controlled by comparing the estimated measure of the association before and after adjusting for confounding. In other words, the measure association was computed both before and after adjusting for the potential confounding factors. The difference between the two measures of association was 4% which is less than 10% and this indicates that there was little and insignificant confounding. The formula used for computing confounding is given by.

Magnitude of confounding = $\frac{{OR}_{crude}-{OR}_{adjusted}}{{OR}_{crode}}$, where OR stands for odds ratio.

Effect modification/ interaction effect occurs when the effect of a factor is different for different groups. The existence of effect modification was tested by considering the crude estimate of the association (odds ratio). The result indicates that there was very close to a weighted average of group-specific estimates of the association. In the current investigation, both stratum-specific estimates of the odds ratio went to one side of the crude odds ratio. Hence, the result of this study indicates that the crude odds ratio was less than the estimates of the odds ratio for the stratum-specific estimates which is an evidence for nonexistence of effect modification.

Multicollinearity in regression analysis occurs when two or more explanatory variables are highly correlated to each other, such that they do not provide unique or independent information in the regression model. Multicollinearity was tested in the current investigation using a metric known as Variance Inflection Factor (VIF). The VIF measures the correlation between predictor variables. The result in this regard revealed that there was no multicollinearity issue.

## Results

### Descriptive statistics

The baseline characteristics of covariates are indicated in Table 1. In Table 1, it is indicated that about 15,400 breastfed women of reproductive age with babies were included in this study. Among these, about 27.6% of the participants were censored, where the actual duration of breastfeeding is unknown and the remaining were events with known breastfeeding duration, 65.7% lived in rural areas, about 42.6% of the women were from poor households, 18.2% of them were from middle-income households and the rest were from high-income households. Table 1 also indicates that about 63.6% of the participants were women with female children and 65.4% of the participants had no job (unemployed). Out of 10,072 unemployed women, 27.7% of them were still breastfeeding and 72.3% of women stopped breastfeeding (event). Of a total of 5328 employed women, 27.5% of the women were still breastfeeding and 72.5% of women stopped breastfeeding.

Among the participants, about 92.1% were non-smokers, 17.9% of the women attained secondary and above education, 49.7% of the women attained primary education, and the rest were uneducated women. The religion category in Table 1 indicates that about 41.1% of the participants were Orthodox, 39.4% were Muslim, and 17.9% of them were Protestant. Regarding their regions, about 11.8% of them were from Addis Ababa city, 12.0% from Oromia, 11.7 from the Old SNNP, 11.0% from Amhara, about 7.1% of them were from Afar, 7.2% from Benishangul- Gumuz, 7.3% from Dire Dawa, 6.5% from Gambela, 5.8% from Harari, and about 10.8% of them were from Tigray region.

### Non-parametric survival analysis

**Fig. 1:**
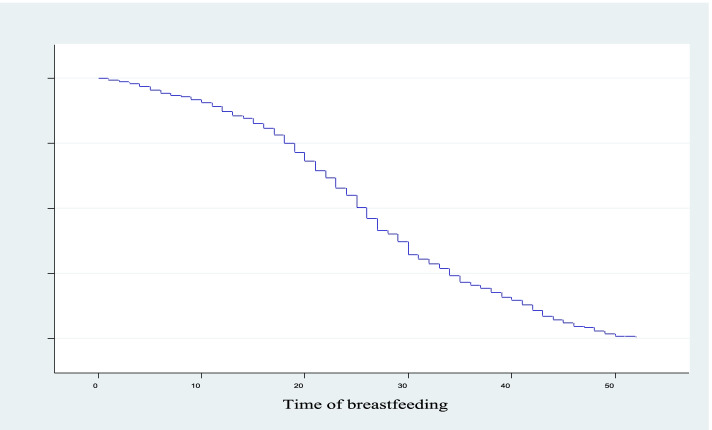
Survival plot for the duration of breastfeeding

**Fig. 2:**
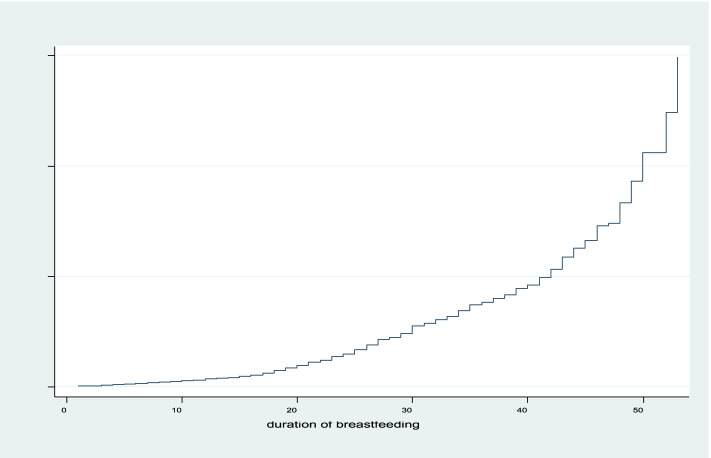
Hazard plot of duration of breastfeeding

The non-parametric analysis was conducted using plots of the Kaplan Meir curves to the survival and hazard experience for the duration of breastfeeding as shown in Fig. 1 and Fig. 2 respectively. The result revealed that the survival plot decreased at an increasing rate at the beginning and decreased further from time to time. This implies that most women breastfed highly soon after birth. On the other hand, the hazard plot increased at an increasing rate at the beginning and increased as time increased.

#### Log-rank test

The log-rank test was used at a 5% level of significance to validate the differences in the survival time of each factor. The difference between the probabilities of an event occurring at any time point was the null hypothesis that has been tested.

#### Cox proportional hazard regression model

After making a comparison of the survivorship experience among groups of covariates, the next important step was model development. At the initial step, in the model-building process was conducted to identify sets of explanatory variables that had the potential for being included in the linear components of a multivariable proportional hazards model. In doing this, Cox proportional hazards regression model, including the univariable Cox proportional hazards models were fitted. From the univariate analysis; place of residence, wealth index of the family, sex of the child, child age, smoking status of women, birth interval, place of residence, wealth index, and women's education level were statistically significant for the variable of interest.

### Model diagnosis for Cox proportional hazards mode1

In the current investigation, the two basic assumptions of the Cox regression model, log-linearity and proportional hazards were tested as shown in Table 2. The log-linearity test revealed that the relationship between log hazard or log cumulative hazard and a covariate was linear. The proportional hazard test in this investigation indicates that the ratio of the hazard function for two individuals with different regression covariates does not vary with time.

**Table 2:** Result of test of proportionality assumption for each covariate

Variable	rho	Chi-Square	Pr > Chi-Square
Wealth index	0.010	8.1129	0.0044
age of women	-0.001	0.4039	0.5251
religion	0.060	11.14	0.0035
Sex of child	0.001	34.56	0.9396
Child age	-0.004	484.5396	0.0001
smoking status	0.269	370.4742	0.0001
place of residence	-0.025	15.1925	0.0001
women education	0.125	93.860	0.0001
Global test	Wald-chi-square = 874.1111 with degree freedom 8, *p*-value < 0.01		

The global fit test in Table 2 also shows that the Wald chi-square test statistic was significant which indicates that the proportional hazards assumption is violated. In other words, the plots of the duration of breastfeeding covariates were not parallel to each other.Thus, there is a violation of the proportional hazards assumption. This indicates that the residuals were not random, there is a systematic pattern and the smoothed plot looks not a straight line and there is some departure from the horizontal line. Thus, there is a violation of the proportional hazards assumption.

Since the proportional hazard assumptions were not satisfied, the accelerated failure time (AFT), including the univariable and multivariable analysis, the model should be conducted for the current data analysis. The univariable analyses were fitted for every covariate, considering AFT models for baseline characteristics of participants. For the duration of breastfeeding data, AFT models of Weibull, exponential, log-logistic, and log-normal distribution were considered. To select the best model for the current analysis, AFT models namely, Weibull, exponential, log-logistic and log-normal distributions were compared using AIC and BIC considering the model with the smallest AIC and BIC is the one that fits the data well, as shown in Table 3. Table 3 indicates that the Weibull distribution had the smallest AIC and BIC. Hence, it has been selected for univariate and multivariate data analysis in the current investigation.

**Table 3:** Comparison of AFT models using AIC, BIC criteria for duration of breastfeeding data

distribution	AIC	BIC
Exponential	29,672.82	29,779.60
Weibull	***19,865.06***	**19,997.29**
log-logistic	20,063.76	20,178.13
log-normal	2,006,372	20,178.13
generalized gamma	19,883.78	20,005.78

*AIC* Akaike’s information criteria, *BIC* Bayesian information criterion

In all univariable analyses of Weibull AFT models, age of women, smoking status, place of residence, wealth index of the household, sex of the child, women's education level, age of children, and birth interval of women were significantly associated with the duration of breastfeeding at 5% level of significance. The summary of the univariable analysis is given in Table 4. Variables that were significant at 5% of the level of significance in the univariable model were included in the multivariable data analysis. The multivariable data analysis under this study is indicated in Table 5. In Table 5, the main effect of the covariates namely place of residence, age of women, birth interval, women’s level of education, smoking status, and age of the child were considered as potential predictors for the variable of interest. The multivariable data analysis with the Weibull AFT models and corresponding AIC and BIC values were conducted as indicated in Table 5.

**Table 4:** Univariable data analysis for Weibull ATF model

Variable	Estimate( ^^^)	SE( ^^^)	Φ	(95% CI ϕ)	*p*-value
**Residence place(Ref = rural)**					
urban	-0.146	0.019	0.86	[0.82,0.90]	0.000*
**Wealth Index(ref. = poor)**					
Meddle	-0.003	0.027	0.10	[0.05,0.15]	0.827
Rich	-0.041	0.021	0.96	[0.94,0.98]	0.000*
**Employment status(Ref. = unemployed**					
Employed	-0.001	0.020	0.10	[0.098,0.102]	0.950
**Women Education level(Ref. = Secondary and above**					
Non-educated	0.087	0.029	1.09	[1.05,1.12]	0.000*
Primary educated	0.175	0.026	1.19	[1.16,1.22]	0.000*
Sex(Ref. = male)					
Female	0.038	0.035	1.04	[1.01,1.08]	0.000*
**smoking status(Ref. = yes)**					
No	0.497	0.035	1.64	[1.58,1.70]	0.000*
**Husband Education level(Ref. = no education)**					
Primary	0.009	0.348	1.01	[0.33,1.69]	0.237
secondary	0.007	0.089	1.01	[0.84,1.18]	0.06
higher	0.019	0.278	1.02	[0.48,1.56]	0.075
**Religion(Ref. = Orthodox)**					
Catholic	-0.013	0.128	0.99	[0.74,1.24]	0.819
Protestant	0.039	0.026	1.04	[0.99,1.09]	0.135
Muslin	0.015	0.021	1.02	[0.98,1.06]	0.100
Traditional	0.069	0.161	1.07	[0.93,1.21]	0.329
Other	0.034	0.172	1.03	[0.69,1.37]	0.653
**birth interval(Ref. = 2–3 years)**					
< 2 years	-0.004	0.045	1.00	[0.91,1.09]	0.052
> = 4 years	0.022	0.025	1.02	[1.00,1.07]	0.000*
Child age	0.02	0.0013	1.02	[1.00,1.07]	0.000*

ϕ Indicates Acceleration factor;* significant at 5% level

*CI* Confidence interval

**Table 5:** The multivariable data analysis result in the final Weibull AFT model

variable		Φ	95 CI for ϕ	SE( ^^^)	*P*-value
**place of residence(Ref. = rural)**					
Urban	*-0.06*	0.94	[0.92,0.96]	*0.009*	0.000*
**women age(Ref. = 15–19)**					
19–24	0.03	1.03	[0.95,1.11]	0.039	0.415
25–29	0.06	1.06	[1.03,1.09]	*0.017*	0.000*
30–34	0.02	1.02	[1.01,1.04]	0.012	0.013*
35–39	0.03	1.03	[1.01,1.05]	0.011	0.007*
40–44	0.003	1.01	[1.01,1.03]	0.011	0.001*
**women education(Ref. = educated women)**					
Non-educated	*0.03*	1.03	[1.00,1.06]	0.013	0.018*
Primary educated	*0.13*	1.14	[1.12,1.16]	0.011	0.000*
**wealth index(Ref. = poor)**					
Middle	*-0.01*	0.99	[0.97,1.01]	*0.012*	0.400
rich	*-0.06*	0.94	[0.92,0.96]	*0.009*	0.000*
**Sex of child(Ref. = male)**					
female	*-0.02*	0.98	[0.96,1.00]	0.009	0.057
**Smoking status(Ref. = Nonsmoker)**					
Smoker	*0.47*	1.60	[1.57,1.63]	0.015	0.000*
age of child	*-0.01*	0.99	[0.89,0.99]	0.001	0.000*
**Birth interval(Ref. = 2-3 years)**					
< 2 year	0.02	1.02	[0.99,1.04]	0.014	0.057
> 4 year	0.01	1.01	[100,1.02]	0.006	0.000*

Remark: ϕ Indicates Acceleration factor;* significant at 5% level

*CI* Confidence interval*, SE* Standard error*, Ref* Reference

*P* = 2.40, 1/p = 0.42,AIC = 19,865.06,BIC = 19,997.29

The result of the Weibull AFT model in Table 5 indicates that level of education, age of women, birth interval, place of residence, sex of the child, smoking status, and wealth status were statistically significant variables for the variable of interest.

### Analysis and model comparisons for parametric shared frailty model

To test the effect of regions on the variable of interest, the multivariable survival analysis was conducted including the Gamma shared frailty term, This was performed using the covariates; place of residence, education level of women, religion, employment status, age of the child, sex of a child, wealth index, women’s level of education, smoking status, and birth interval. In this study, the AIC and BIC criteria were considered to compare various candidates of parametric shared frailty models considering, the model with the smallest AIC and BIC as the best model.

The parametric baseline distributions namely Gamma frailty distribution, log-normal, and Inverse Gaussian distributions were fitted and compared by considering the regions of the women as frailty terms. The effect of random component (frailty) was significant for Weibull gamma shared frailty because of its smallest AIC and BIC values [20]. The final Weibull gamma shared frailty model is indicated in Table 6.

**Table 6:** Parametric estimates of Weibull -Gamma Frailty Model

Covariate	Estimate	Φ	95% CI for ϕ	*SE*	*P*-value
**place of residence(Ref. = rural)**					
Urban	-0.04	0.96	[0.94,0.97]	0.001	0.001*
**Women’s age(Ref. = 15–19)**					
19–24	0.01	1.010	[1.001, 1.05]	0.039	0.010*
25–29	0.02	1.020	[1.02, 1.40]	0.018	0.001*
30–34	0.25	1.284	[1.06, 1.41]	0.012	0.020*
35–39	0.03	1.030	[1.01, 1.55]	0.011	0.005*
40–44	0.04	1.041	[1.01, 1.22]	0.011	0.019*
**Level of education(Ref. = secondary and above)**					
Non-educated	0.03	1.03	[1.00,1.06]	0.014	0.039*
Primary	0.12	1.13	[1.11,1.15]	0.011	0.000*
**Wealth index(Ref. = Poor)**					
Middle	-0.03	0.97	[0.93,0.99]	0.012	0.022*
rich	-0.07	0.93	[0.91,0.95]	0.010	0.000*
**Sex of child(Ref. = male)**					
Female	-0.02	0.98	[0.96,1.00]	0.009	0.091
**Smoking status(Ref. = yes)**					
No	0.47	1.60	[1.57,1.63]	0.015	0.000*
child age	-0.01	0.99	[0.76, 0.99]	0.001	0.000*
**birth interval(Ref. < 2 years)**					
< 2–3 years	0.02	1.02	[1.09, 1.25]	0.014	0.027*
> 3 years	0.25	1.28	[1.06,1.43]	0.006	0.000*

ϕ Indicates Acceleration factor;* significant at 5% level

*CI* Confidence interval*, SE* Standard error*, Ref* Reference*, λ Scale*

Table 6 indicates that the residential area had a significant effect on the duration of breastfeeding. Hence, the expected duration of breastfeeding for urban women was decreased by 4% as compared to rural women, keeping the other covariates constant(Φ = 0.96; 95% CI; (0.94,0.97); *p*-value = 0.001). This indicates that women in rural had a prolonged duration of breastfeeding than urban women.

The level of education had a significant effect on the variation in the duration of breastfeeding. Hence, comparing non-educated women with secondary and above, the expected duration of breastfeeding for non-educated women was increased by 3% as compared to secondary and above women keeping the other covariates constant(Φ = 1.03; 95% CI; (1.00,1.06); *p*-value = 0.039). Similarly, the expected duration of breastfeeding for primary educated women was increased by 13% as compared to secondary and above, keeping the other covariates constant(Φ = 1.13; 95% CI; (1.11,1.15); *p*-value < 0.001). This result indicates that the more educated women had a short duration of breastfeeding as compared to non-educated or less educated women.

The age of a child also played a significant role in the variation in the duration of breastfeeding. Hence, as the age of a child increased by one month, the expected duration of breastfeeding decreased by 1% keeping the other covariates constant (Φ = 0.99; 95% CI; (0.76, 0.99); *p*-value < 0.001). Hence, the increase in the age of a child leads to a decrease in the duration of breastfeeding.

The smoking status of mothers/women also played a significant role in the variation in the duration of breastfeeding. Comparing smoker women with non-smoker one, the expected duration of breastfeeding for non-smoker women was increased by 60% as compared to smoker women, keeping the other covariates constant. (Φ = 1.60; 95% CI; (1.57, 1.63); *p*-value < 0.001).

The birth interval between consecutive births had also a significant effect on the variation in the duration of breastfeeding. Hence, comparing a woman whose birth interval, is between 2–3 years with that of less than 2 years, the expected duration of breastfeeding for a woman whose birth interval, is 2–3 years was increased by 2% as compared to a woman whose birth interval, < 2 years, keeping the other conditions constant (Φ = 1.02; 95% CI; (1.09, 1.25); *p*-value < 0.027). Similarly, the expected duration of breastfeeding for a woman whose birth interval > 4 years was increased by 28% as compared to a woman whose birth interval < 2 years (Φ = 1.28; 95% CI; (1.06, 1.43); *p*-value < 0.01).

The age of women was also another significant variable for the variation of the duration of breastfeeding. Hence, the expected duration of breastfeeding for a woman whose age, 40–44 years was increased by 4% as compared to a woman of age, 15–19 years, given that the other covariates were constant (Φ = 1.041; 95% CI; (1.01, 1.22); *p*-value = 0.019) and the expected duration of breastfeeding for a woman of age,35–39 years was increased by 3% as compared to a woman whose age, 15–19 years (Φ = 1.030; 95% CI; (1.01, 1.55); *p*-value = 0.005). Hence, the more the age of the women leads to the longer duration of breastfeeding for the current investigation.

## Discussion

Place of residence has a significant role in the variation of the time-to-breastfeeding in Ethiopian women of reproductive age with babies. Women who lived in urban areas had a shorter duration of breastfeeding than women who lived in rural areas. Rural inhabitants have usually no access to getting a different alternative to feed their children [21]. The only option is the feeding of breast milk which may result in a longer duration for rural women as compared to urban. This result is supported by another previous study [22]. Most of the time rural women are housewives and they have a greater chance to get their child a day to give their breast milk.

A woman's age has a significant effect on the variable of interest. Women of older age have a longer duration of breastfeeding. This finding is supported by another study [23]. One of the previous studies reported that aged women are highly associated with a longer duration of breastfeeding [24]. The potential reason for this might be that woman in the menopause period has less chance of getting an extra child and she needs to extend her breastfeeding. Most of the time, young women, who made sexual intercourse, may have an extra child and they may be forced to stop her breastfeeding for the current child to prepare for the newly coming child [25].

Women's level of education has a significant effect on the variation of the duration of breastfeeding of Ethiopian women of reproductive age with babies. Educated women have a shorter duration of breastfeeding than non-educated or less educated women. The potential reason for this might be the fact that more educated women are government employed and they are forced to continue their regular time work for a short period (four months) after birth. This result is supported by many other previous studies [26–28]. Previously conducted research states that illiteracy has a positive association with the duration of breastfeeding [29].

The wealth index had a significant association with the duration of breastfeeding. Women of rich wealth status families had a shorter duration of breastfeeding than poor wealthy status. The potential reason for this might be the fact that rich women used another alternative for feeding their children. This finding is supported by another study [30]. It is reported that low-income status was associated with a longer duration of breastfeeding.

The smoking status of women has a significant effect on the duration of breastfeeding. Smoker women have a shorter duration of breastfeeding than non-smokers [31]. The potential reason for this might be that smoker women have lower milk production and let-down problems; this may be related to poor lactation management rather than physiological causes. And also smokes immediately after breastfeeding to cut down on the amount of nicotine in her milk during nursing [32]. The other reason might be the fact that smoker women may not have a strong physiological mechanism. This finding is supported by another previously conducted study [33]. Another similar study reported that smoking has a negative effect on the duration of breastfeeding [34].

The birth interval had a significant association with the duration of breastfeeding. Hence, women with a birth interval of 2–3 years had a longer duration of breastfeeding as compared to those women with a birth interval < 2 years. Furthermore, women with more than three years of birth interval have a longer duration of breastfeeding as compared to those women with breath intervals of fewer than two years. Mothers with short period birth intervals should give their milk to the newly coming child rather than the previous. This finding was supported by [35], who reported that breastfeeding was highly associated with birth interval.

## Conclusion

The result of Weibull AFT and Weibull-gamma frailty models revealed that place of residence, age of women, level of women’s education, age of the child, smoking status, birth interval, and wealth of household was found to be significant predictors of the duration of breastfeeding of women of reproductive age with babies in Ethiopia. Among these significant predictors, the age of women, and the birth interval of consecutive births had a positive association with the duration of breastfeeding. On the other hand, the level of education, wealth status of women, smoking status of women, and urbanization had a negative association with the duration of breastfeeding.

In the current study, the clustering effect was significant (*p*-value = 0.000) in Weibull-gamma shared frailty model. This indicates that there was heterogeneity between the regions on the duration of breastfeeding. Weibull-gamma frailty model was in favor of the Weibull AFT model and well fitted the duration of breastfeeding data. The estimated median survival duration of breastfeeding for Ethiopian women of reproductive age with babies was found to be 23 months. The median time of duration of breastfeeding for rural women was 26 months. The concept behind Weibull–the gamma shared frailty model indicates that there was a frailty (clustering) effect on the duration of breastfeeding. Hence, regions played a significant role for the presence of heterogeneity and necessitate the use of shared frailty models.

### Limitation of study

This research was not without limitation; one of the limitations of current investigation whether the mothers delivered by cesarean or spontaneous was not identified. Hence, the duration of breast feeding by mode of delivery was not considered. Mainly mothers who delivered by cesarean section may fed her baby for shorter duration than mother delivered spontaneously.

As a recommendation, the health staff and those concerned bodies should encourage and advise women of childbearing age to prolong the duration of breastfeeding. The breastfeeding-promotion programs in Ethiopia should give special attention to young mothers, mothers who have a short duration (educated mothers, urban mothers, mothers with high wealth index, and smoker mothers). The health-related education and awareness creation should be conducted by the health staff about the importance of elongating the duration of breastfeeding. Further investigation, with the inclusion of additional variables not included here such as mode of delivery of women, are recommended to have additional information on factors affecting the duration of breastfeeding in the study area. Further studies should be also conducted in each region of Ethiopia and identify other factors that are not included in this study.
